# A highly sensitive detection for foot-and-mouth disease virus by gold nanopariticle improved immuno-PCR

**DOI:** 10.1186/1743-422X-8-148

**Published:** 2011-03-31

**Authors:** Yao-zhong Ding, Yong-sheng Liu, Jian-hua Zhou, Hao-tai Chen, Gang Wei, Li-na Ma, Jie Zhang

**Affiliations:** 1 State Key Laboratory of Veterinary Etiological Biology, Key Laboratory of Animal Virology of Ministry of Agriculture, Lanzhou Veterinary Research Institute, Chinese Academy of Agricultural Sciences, Lanzhou, 730046, Gansu, China; 2 lanzhou Jiaotong Univesity Bowen College, Lanzhou, 730101, Gansu, China

## Abstract

**Backgroud:**

Foot-and-mouth disease (FMD) is one of the most contagious of all artiodactyl animal diseases, and its infection has an obvious ability to spread over long distances and to contribute to epidemics in FMD-free areas. A highly sensitive and specific method is required to detect FMDV. In this study, we evaluated the usefulness of a bio-barcode assay (BCA) technique for detecting clinical samples of FMDV.

**Methods:**

Highly sensitive gold nanopariticle (GNP) improved immuno-PCR (GNP-IPCR) which derived from the bio-barcode assay (BCA) was designed for the detection of FMDV. The target viral particles were captured by a polyclonal antibody coated on ELISA microplate, followed by adding GNP which was dually modified with oligonucleotides and a FMDV specific monoclonal antibody (MAb) 1D11 to form a sandwiched immune complex. After the formation of immuno-complex, the signal DNA was released by heating, and consequently characterized by PCR and real time PCR.

**Results:**

The detection limit of GNP-PCR could reach to 10 fg/ml purified FMDV particles, and the assay can detect clinical samples of FMDV with highly sensitivity, while detect limit of conventional ELISA is 100 ng/ml in this study.

**Conclusion:**

GNP-IPCR may provide a highly sensitive method for the detection of FMDV.

## Backgroud

Foot-and-mouth disease virus (FMDV), which belongs to the *Aphthovirus *genus of the family *Picornaviridae*, is a single-stranded positive sense RNA virus of 8,500 bp and is enclosed by an icosahedral capsid [1]. The virus exists as seven serologically distinct types (O, A, C, Asia 1, SATs 1, 2, 3) [2]. The virus affects artiodactyl, especially cattle and swine, and FMD is one of the most contagious of all animal diseases. In 2001, outbreaks of FMD in Taiwan, South Korea and the United Kingdom resulted in the slaughter of millions of animals and huge economic losses [3]. This virus can survive in meat and other animal products for a long periods [4], and FMD is endemic in large area of South America, Asia, Africa, and its infection has an obvious ability to spread over long distances and to cause epidemics in FMD-free areas.

Many methods have been developed to detect the FMDV, such as ELISA [5], hybridization assays [6], conventional RT-PCR [7] and real-time RT -PCR [8-14]. Generally, the sensitivity of ELISA is lower, but which can detect antigens or antibodies. The sensitivity of PCR is higher, but it can only detect DNA/RNA of the virus, and can also lead to false-positive results.朗读

Recently, a new highly sensitive assay, namely bio-barcode amplification (BCA) assay, was developed for ultrasensitive detection of target proteins and nucleic acids, the detection limit could reach the level of 30 attomolar (aM) [15-17]. BCA combines the advantages of both ELISA and PCR, it has not only the sensitivity of PCR, but also can detect the antigens or antibodies. In the BCA, gold nanoparticles (GNPs) could conjugate with DNA and specific monoclonal antibody (Mab). Many DNA immobilized on the surface of GNP would further improve the sensitivity of conventional immuno-PCR [18,19]. In this study, the application of BCA was evaluated for detecting clinical FMD samples.

## Materials and methods

### Cells and virus

BHK-21 cells were grown in MEM medium (Qingdatianyi Co., China) containing 4% newborn calf serum (PPA, Australia), and used to replicate FMDV. FMDV strain China/99 specific MAb 1D11 and the polyclonal antibody rabbit sera against the FMDV type O used in this study were provided by our laboratory.

Real MasterMix (SYBR GREEN) kit was provided by TaKaRa Biotechnology Co., Ltd. (Dalian, China) and rabbit anti-guinea pig IgG which was conjugated with horseradish peroxidase (HRP) was provided by Sigma (Sigma, US).

GNPs with an average diameter of 30 nm were obtained from Ted Pella Inc (CA, USA). All oligonucleotides and primers were synthesized from Sangon Co., Ltd. (Shanghai, China) (Table 1).

**Table 1:** Oligonucleotides and primer sequences used in this work.

name	Sequence
Capture DNA	SH-5'-dA15TTCATCGCCCTTGGACTACGACTCTGACTGATCGCTAAATCGTG-3'
Signal DNA	5'-CACGATTTAGCGATCAGTCAGAGTCGTAGTCCAAGGGCGATGAA-3'
F-signal DNA	FAM-5'-CACGATTTAGCGATCAGTCAGAGTCGTAGTCCAAGGGCGATGAA-3'
Forward primer	5'-CATCGCCCTTGGACTACGA-3'
Reverse primer	5'-CACGATTTAGCGATCAGTCAGAG-3'

*dA_15 _means that there are 15 dA, and SH means that this end of the oligonueotide is modified by alkanthiol group.

### Preparation of bi-functionalized GNP conjugated with oligonucleotides and monoclonal antibodies

GNPs bi-functionalized with MAb 1D11 and the conjugation DNA were prepared as reported previously [15]. First, the primary gold nanoparticles solution was adjusted to pH 9.2 with 1 M NaOH. Then, 1 ml of the solution was mixed with 6 μg MAb 1D11 at room temperature and was mildly shaking for 30 min. This solution was added with 1 OD thiolated conjunction DNA for 16 h at 10°C. Then, a procedure of salt-aging was carried out by adding 2 M NaCl in a six-stepwise-addition within 24 h to this solution in order to make 0.1 M of the final concentration of NaCl. 0.3 ml of a 10% bovine serum albumin (BSA) solution was added into the solution with extensive incubation at room temperature for 30 min to stabilize and achieve passivation of GNPs. The solution was centrifuged at 4°C for 20 min, and the supernatant was removed. The nanoparticles were resuspended in 0.1 M phosphate buffered solution (PBS, pH7.4) and centrifuged again in order to achieve further purification of nanoparticles. The nanopaticles were resuspended in the 400 μl PBS with 1 OD of the signal DNA and hybridized at 37°C for 1 h. The similar centrifugation procedure was performed to remove the redundant signal DNA and obtain the bi-functionalized GNPs. Finally, the probes were resuspended in PBS with 0.01% Tween-20 (v/v) and 0.1% BSA (w/v). This bi-funtionalized GNPs probes solution was stored at 4°C.

### Assay for FMDV by ELISA

Conventional ELISA was performed as reported previously [20]. Each microplate (Corning Costar Inc., US) were coated with polyconal antibody (10 μg/ml) in 0.1 M carbonate/bicarbonate buffer (pH 9.4) overnight at 4°C. Plates were blocked with 5% skim milk in PBS and washed three times with PBST (containing 0.1% Tween-20, pH7.4). After three times of washing, each microplate was added with 50 μl of tenfold serial dilutions of the purified FMDV (1 ug/ml to 0.1 pg/ml) incubated for 1 h at 37°C. The plates were subsequently washed, then 50 μl MAb 1D11 (1:1000 dilution) was added and incubated for 1 h at 37°C. After three washings, 50 μl Goat anti-mouse IgG which was conjugated with horseradish peroxidase (HRP) (Sigma) was added and incubated for 1 h at room temperature. Three time of washing with PBST, the ELISA reaction was developed by the addition of ophenylenediamine-H_2_O_2 _for 20 min at 37°C. The reaction was stopped by the addition of 50 μl 1.5 M H_2_SO_4 _and the microplate was read at OD _490 _by a plate reader (Bio-Rad 680, USA).

### Detection of FMDV by GNP probe

All procedure of coating and blocking for GNP-IPCR were carried out by same way with the ELISA. The assay of GNP-IPCR was conducted as described previously [19]. The microplates were washed using 0.05 M PBSET (including 5 mM EDTA and 0.05% Tween-20). 50 μl of tenfold serial dilutions of purified FMDV (10 pg/ml to 0.01 fg/ml) were respectively added into the microplates and then incubated at 37°C for 1 h, following by five times of washing. Then 50 μl GNP probe was added into microplates and incubated for 1 h at 37°C, washed five times with PBS, add 50 μl of double distilled H_2_O into each microplates. Finally, the plates were handled in a water bath at 80°C for 10 min to release the signal DNA which is then analyzed by PCR.

PCR amplification was performed by adding 5 μl of the free signal DNA as templates (0.2 μM each primers, 1 mM dNTP, 10 × buffer and 2.5 U Taq DNA polymerase). The amplification products were analyzed by 2% agarose gel electrophoresis.

### Detection of Real-time PCR for signal DNA

Real time PCR amplification was performed by adding 5 μl of the released signal DNA solutions as templates, added 0.5 μl forward primer (10 μM) and reverse primer (10 μM) listed in Table 1 into the MasterMix (SYBR GREEN) kit in a total volume of 20 μl. The cycling parameters were an initial DNA denaturation step at 95°C for 30 sec followed by 40 cycles of PCR with DNA denaturation at 95°C for 15 sec and primer annealing and extension at 68°C for 30 sec.

### Detection Clinical FMDV samples using GNP-IPCR

To evaluate whether this method is perfect for clinical FMDV sample assays, a total of 30 sera, 20 vesicle fluids of type O of FMDV and 26 negative sera of FMDV and 10 type Asia 1 of FMDV samples were isolated in our laboratory to be identified by GNP-IPCR.

## Results

### Detection FMDV Antigen using GNP-IPCR and ELISA

**Figure 1:**
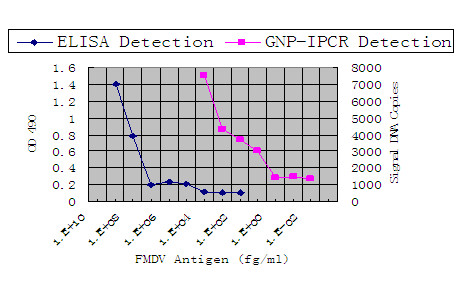
**Detection of serial dilutions of purified FMDV using GNP-IPCR and ELISA**. The detect limit of ELISA was 100 ng/ml purified FMDV and that of GNP-IPCR was 10 fg/ml.

To compare the sensitivity and detection limit between the GNP-IPCR and ELISA, we applied both GNP-IPCR and ELISA to detect the purified FMDV antigens. As shown in Figure 1, the detection limit of ELISA is 100 ng/ml, but the detection limit of GNP-IPCR is about 10 fg/ml of FMDV purified antigens, which was 7 titers of magnitude more sensitive than general ELISA system.

### Detection of FMDV by GNP-IPCR real-time PCR

**Figure 2:**
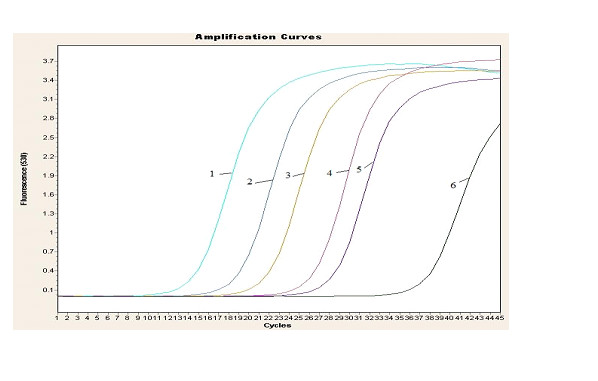
**SYBR-GREEN real time PCR detection of FMDV based on GNP-IPCR**. Curve 1-5 represents samples with FMDV concentration of 10 pg/ml, 1 pg/ml, 100 fg/ml, 10 fg/ml and 1 fg/ml, and 6 represent negative control, respectively.

Gel electrophoresis is a wildly used tool in immuno-PCR to quantify the PCR amplification, but it has some shortcoming such as time consuming. Here, a SYBR green-based fluorescence quantification RT-PCR was adopted for analysis of the released signal DNA. As shown in Figure 2, clear distinctness in signals was found between FMDV samples and the negative control. The FMDV concentration ranges from 10 pg/ml to 1 fg/ml, and 1 fg/ml purified FMDV can be distinguished from the negative control by this assay.

### Detection FMDV Clinical samples using GNP-IPCR

Detection of FMDV clinical sample using GNP-IPCR indicated that 30 sera, 20 vesicle fluids of type O of FMDV was identified as positive, 1 negative serum identified as suspected positive may be due to the sample contamination, no cross reaction of this assay with serotype Asia 1 (Table 2). Owing to its rapidity, sensitivity and specificity, GNP-IPCR assay is suitable for clinical diagnosis and surveillance of FMDV, and the whole assay could be completed within 4 h.

**Table 2:** Evaluation of 85 clinical samples by GNP-IPCR

Specimen	**Sample No**.	No. (%) of positive samples by GNP-ELISA
Serum of type O	30	30 (100)
Vesicle fluid of type O	20	20 (100)
Serum of type Asia 1	6	0 (0)
Vesicle fluid of type Asia1	4	0 (0)
Negative serum	26	1 (3.4)

## Discussions

ELISA and conventional PCR are standard laboratory assays which applied to detect FMDV [21-25]. These assays have been shown restricted on the detection of FMDV antigen [14] because the ELISA lack of the high sensitivity, and the PCR can only detect DNA/RNA 朗读. To combine the applicability for detection of antigen of ELISA and the high sensitivity of PCR, Sano et al., developed the immuno-PCR (IPCR) method [26]. This application is also limited for the lack of standards related products and difficulty of synthesis of antibody-oligonucleotides conjugation of IPCR. We are pay attention to the ability of the BCA-IPCR for detecting FMDV. This method offers an innovative approach to detect target proteins that overcomes shortcomings of IPCR. The method is exquisitely specific because antigens or antibodies bound to GNPs and the GNPs are directed against two or more distinct epitopes [27-29], and the method is more convenient and time-saving than conventional IPCR assays [30-33].

BCA technology combines the advantages of PCR and ELISA which have a specificity and sensitivity of detection of clinical samples. The detect limit of GNP-PCR can be 10 fg/ml purified FMDV samples while detect limit of conventional ELISA is 100 ng/ml in this study. The detect limit of this study was lower than the theoretical detect limit. This may be due to the influence of some factors, for example, the combination efficiency of the monoclonal antibody, the captured efficiency of the antigen, or the sample contamination [34]. Meanwhile, the results of detection clinical FMDV samples indicated that GNP-IPCR assay is suitable for clinical diagnosis and surveillance of FMDV. To our knowledge, this is the first report that GNP-PCR was applied in the detection of FMDV antigen.

## Competing interests

The authors declare that they have no competing interests.

## Authors' contributions

JZ, YD and YL designed the study and drafted the manuscript. JZ, HC, GW and LM prepared the monoclonal antibodies and performed GNP-IPCR. All authors read and approved the final manuscript.
